# Natriuretic Peptides in the Cardiovascular System: Multifaceted Roles in Physiology, Pathology and Therapeutics

**DOI:** 10.3390/ijms20163991

**Published:** 2019-08-16

**Authors:** Speranza Rubattu, Massimo Volpe

**Affiliations:** 1 Department of Clinical and Molecular Medicine, School of Medicine and Psychology, Sapienza University of Rome, 00189 Rome, Italy; 2 IRCCS Neuromed, 86077 Pozzilli (Isernia), Italy

**Keywords:** natriuretic peptides, arterial hypertension, pulmonary arterial hypertension, heart failure, stroke, atrial fibrillation, ARNi, MANP

## Abstract

The natriuretic peptides (NPs) family includes a class of hormones and their receptors needed for the physiological control of cardiovascular functions. The discovery of NPs provided a fundamental contribution into our understanding of the physiological regulation of blood pressure, and of heart and kidney functions. NPs have also been implicated in the pathogenesis of several cardiovascular diseases (CVDs), including hypertension, atherosclerosis, heart failure, and stroke. A fine comprehension of the molecular mechanisms dependent from NPs and underlying the promotion of cardiovascular damage has contributed to improve our understanding of the molecular basis of all major CVDs. Finally, the opportunity to target NPs in order to develop new therapeutic tools for a better treatment of CVDs has been developed over the years. The current Special Issue of the Journal covers all major aspects of the molecular implications of NPs in physiology and pathology of the cardiovascular system, including NP-based therapeutic approaches.

The natriuretic peptides (NPs) family includes a class of hormones [atrial (ANP), B-type (BNP) and C-type (CNP)] and their receptors [natriuretic peptide receptor-A (NPRA), receptor-B (NPRB), and receptor-C (NPRC)] needed for the physiological control of cardiovascular functions. First, the discovery of NPs provided a fundamental contribution for the understanding of the physiological regulation of blood pressure (BP) and of cardiovascular and renal functions [1]. Subsequently, abnormalities of the NPs physiological properties were implicated in the pathogenesis of major cardiovascular diseases (CVDs), such as hypertension and heart failure (HF) [2,3]. Finally, a more thorough comprehension of the molecular mechanisms linked to NPs actions through their distinct receptors has contributed to improve our understanding of key molecular mechanisms of cardiovascular homeostasis, as well as the progression of several CVDs [3,4]. 

As a matter of fact, the NPs system has provided over time a continuous, attractive source of new knowledge and discoveries regarding the pathogenesis, diagnosis, prognosis, and therapy of CVDs. In particular, the opportunity to target NPs in order to design new therapeutic tools for a more effective treatment of CVDs has been developed, ultimately culminating in the introduction of a new class of drugs for the management of HF, the angiotensin receptor neprilysin inhibitor (ARNi) [5,6]. 

The continuous interest in this field of biomedical research is documented by accumulating data produced from several expert scientific groups. This issue of the Journal collects some original and review articles on the molecular and biomedical aspects concerning NPs, with a discussion of their current clinical and therapeutic applications. 

The cellular effects of NPs include the regulation of cell proliferation, angiogenesis, apoptosis, fibrosis, and inflammation [3]. Their anti-proliferative, anti-fibrotic, and anti-hypertrophic effects, including the underlying signaling pathways, were largely documented at both cardiac and vascular levels [3]. In this issue, a review of the literature presented by Forte M. et al. summarizes the current knowledge on the cardiovascular pleiotropic effects of NPs and highlights the most relevant findings that underscore the NPs system as a key player in the cardiovascular remodeling process [7]. A major strength of this aspect of NPs function was initially provided by genetically modified animal models showing that lack of either the ANP (*Nppa*) or NPRA gene (*Npr-1*) led to hypertension and marked cardiac hypertrophy, the latter being independent from high blood pressure levels [7]. In particular, as outlined in the article by Pandey K.N. [8], the gene-targeted (gene-knockout and gene-duplication) mouse models demonstrated the key roles of guanylyl cyclase/NPRA in cardiovascular disease states. Above all, we learned that lack of *Npr-1* led to salt-sensitive increases in BP whereas *Npr-1* gene duplication lowered BP and protected against high dietary salt concentrations [8]. The findings obtained in animal models were subsequently translated to the human disease [3,8]. In fact, both genetic and clinical studies could demonstrate the significant associations of variant alleles at *Nppa*, BNP gene (*Nppb*), and *Npr-1* with cardiovascular disorders in humans [3,8,9]. 

Interestingly, NPs control the lipid metabolism through an anti-lipolytic effect [10]. Of note, they promote mitochondria biogenesis in adipocytes and the process of “browning” of white adipocytes to increase energy expenditure [11]. Herein, a novel original mechanism underlying the anti-lipolytic effect of ANP is presented by Bordicchia M. et al. [12]. This mechanism, supported by experimental in vitro evidence, refers to the inhibition by ANP of Proprotein convertase subtilisin/kexin type 9 (PCSK9), the enzyme responsible for Low Density Lipoprotein (LDL) receptor (LDLr) degradation [13]. Specifically, the original work by Bordicchia M. et al. demonstrates that ANP inhibits PCSK9 expression in human adipocytes, therefore reducing LDLr degradation [12]. It is known that the inhibition of PCSK9, through a specific antibody, allows the accumulation of LDLr and the decrease of LDL cholesterol level in the blood [13]. This strategy has represented a breakthrough of the current therapeutic approaches to treat hypercholesterolemia [14]. By blocking PCSK9 induction, ANP appears to mimick, although to a much lower extent, the action of PCSK9 inhibitors, evolocumab and alirocumab [15,16]. It will be interesting in the future to test LDL cholesterol levels in patients undertaking ARNi and presenting with higher ANP circulating levels [17].

Both the hemodynamic and cellular effects of NPs explain the pathogenetic involvement of NPs in hypertension and related target organ damage. In particular, as discussed in this issue of the Journal, the comprehension of the fine molecular mechanisms underlying hypertension has been largely improved through the dissection of the molecular genetics of the NPs system [18]. Nowadays, genetic variations of *Nppa*, *Nppb*, CNP gene (*Nppc*), *Npr-1*, NPRC gene (*Nprc*), *Corin*, and Proprotein convertase subtilisin/kexin type 6 gene (*PCSK6*) are known contributors to hypertension development in experimental models as well as in humans through a decreased function of the system and of its impact on BP regulation [18]. Furthermore, by dissecting molecular alterations of the NPs system components, we have been able to understand, at least in part, the pathogenesis of cardiovascular damage in hypertension. Most importantly, a harmful variant of human *Nppa* (the T2238C/ANP, rs5065), that is frequently encountered in the general population (14% frequency of the allele variant), has shown functional deleterious properties that completely diverge from those of the wild type form, which makes this molecular variant a significant contributor to cardiovascular acute events such as stroke and myocardial infarction [19]. On the other hand, a protective *Nppa* variant (rs5068) is able to reduce the cardiometabolic risk by increasing the circulating ANP level and its beneficial cardiovascular and metabolic properties [20]. Furthermore, a less frequent *Nppa* variant (rs5063) was associated to reduced left ventricular mass in hypertension [21]. Overall, the experience gained from several research groups with the studies on molecular variants of *Nppa* support the existence of genetic predictors of cardiovascular risk that contribute to the individual risk profile (as part of the emerging field of predictive medicine).

NPs represent today well established and useful diagnostic biomarkers in HF, being of particular help for the differential diagnosis of dyspnea in the emergency room [22]. The increase of amino-terminal (NT)-proBNP/BNP levels reflects the ventricular dysfunction characterizing the condition of HF with reduced ejection fraction (HFrEF), whereas their decrease reliably reflects functional cardiac improvement due to the therapeutic interventions [23]. ANP behaves in a similar manner, although it is not routinely used in clinical practice mainly due to its shorter half-life and lability. The mid-regional amino-terminal ANP (MR-proANP), detected through an immunoassay toward the segment including aminoacids 53-90 of the ANP amino-terminal portion, is a more stable form and offers more specific useful applications [24,25]. Both ANP and BNP also play a prognostic role in HF [26,27]. The accumulation of NPs is not sufficient to maintain a proper hemodynamic balance in cardiac failure, particularly with the progression of the disease. In fact, a state of “resistance to NPs” is described in HF patients, raising the need to increase further their plasma levels in order to achieve a better circulatory homeostasis in cardiac failure [28]. In this issue of the Journal, the role of NPs is discussed in the condition of HF with preserved ejection fraction (HFpEF) [29]. Although with some controversies, lower levels of BNP are found by the majority of the studies in HFpEF [29,30]. Of interest, the significance of MR-proANP in the context of HFpEF is growing as a more specific and more informative marker that parallels the trend of BNP [24]. Therefore, raising the NPs levels is expected to allow an improved hemodynamic profile in HFpEF as well. The upcoming results of the PARAGON trial (that tested the potential benefits of ARNi in HFpEF patients) could soon clarify this important question [31].

HF is often associated to atrial fibrillation (AF), a condition that on its own presents with higher BNP levels [32,33]. This combination raises the need to interpret correctly the level of NPs for both diagnostic and prognostic purposes. The BNP level may not differ between HF patients with AF and HF patients without AF [34]. In fact, higher cut-off levels of BNP need to be taken into consideration to improve the specificity and likelihood of correct diagnosis of HF in the presence of AF [35]. Moreover, as discussed in this issue, the role of NPs in the screening for the new onset of incident AF and for the prediction of AF recurrence after cardioversion and pulmonary vein isolation may reveal useful in the clinical setting [33,36,37]. 

In the context of HF, a renal dysfunction often develops (cardiorenal syndrome). As reviewed in this issue by Okamoto R. et al. [38], BNP is a major player in the heart–kidney connection and it plays important protective roles within the kidneys mainly through its inhibitory effect on the renin-angiotensin system and the sympathetic nervous system. Thus, by promoting diuresis, natriuresis, and vasorelaxation, it counteracts not only HF but also chronic kidney disease (CKD) development. In fact, BNP and NT-proBNP levels are higher in acute HF patients with renal dysfunction as compared to patients with normal renal function [39]. Importantly, it has been shown that BNP infusion may contribute to prevent development of CKD in HF [40].

The strength of the relevance of NPs in HF is reinforced by an interesting review article of this Special issue. Specifically, the article by Cao Z. et al. focuses on the role of NT-proBNP/BNP as valuable diagnostic biomarkers of cardiac dysfunction in deceased individuals [41]. This original observation extends the application of these HF biomarkers to forensic medicine apart from the standardized use in clinical practice. No other biomarker has ever been reported to diagnose cardiac dysfunction postmortem.

An important component of the NPs system is represented by CNP, which acts through either the NPRB or NPRC receptors. CNP is mainly synthetized by endothelial cells and also by cardiomyocytes and fibroblasts. It circulates in the blood at very low amounts, offering a clear example of an autocrine/paracrine mediator within the cardiovascular system [42]. The most recent discoveries regarding CNP functions have been reported in the review article by Moyes A. et al. [43]. These authors underscore novel functions of CNP, such as control of inflammation, angiogenesis, cell proliferation, and anti-atherosclerotic effect in the blood vessel; control of cardiomyocyte contractility, fibrosis, hypertrophy and even of electrophysiological activity of the heart [43]. These multiple functions make CNP a multifaceted paracrine regulator within the cardiovascular system. In the presence of a dysregulation of CNP, the development of CVDs is favored. For instance, since CNP controls BP levels through a potent vasodilation within the microvasculature, abnormalities of CNP function contribute to hypertension development [44]. CNP increases in HF, in parallel to ANP and BNP and to its receptor NPRC, and it correlates to disease severity and outcome [45]. In fact, these observations have focused the attention to CNP as a potential therapeutic target in both hypertension and HF.

Among the recent discoveries regarding the NPs system, the one that deserves particular attention is the potential involvement of NPRC signaling in the pathogenesis of pulmonary arterial hypertension [46]. The article by Egom E. provides a revision of the literature supporting the link between abnormalities of NPRC signaling and pulmonary vascular remodeling, pulmonary fibrosis, and chronic obstructive pulmonary disease [47]. The latter are explained by the disruption of the anti-proliferative effects of NPRC via the Gqα/mitogen-activated protein (MAP) kinase signaling pathway [48]. 

The main therapeutic approaches to treat CVDs involving the NPs system are based on either the development of peptide analogs or the blockade of peptides catabolism [3,4]. In this issue, Cannone V. et al. describe one of the most promising ANP analog, the MANP, a 40 amino acid peptide with a 12 amino acid extension to the carboxyl-terminus of ANP [49]. This peptide analog, that is more resistant to degradation, is progressively gaining more interest for its future application in clinical practice. In fact, it has been tested in both experimental and clinical settings with evidence of a significant prolonged anti-hypertensive effect. Its cardiometabolic protective properties are also being currently investigated in humans [49].

An overview of the strategies aimed at blocking the NPs catabolism through a NPRC blockade, and particularly through NEP inhibition, is presented by Volpe M. et al. [50]. The approach based on NEP inhibition led to the recent development of a new class of drug called ARNi, which currently represents a valuable therapeutic tool for the treatment of HRrEF and may become, in the near future, an essential tool for the treatment strategy toward many other CVDs, possibly also hypertension [5,50]. So far, the only available compound is sacubitril/valsartan.

Overall, the comprehension of the multiple functional roles of NPs, gained over the last 35 years, makes this hormonal system an essential contributor to the maintenance of the cardiovascular health. On the other hand, a deeper understanding of the complex molecular mechanisms underlying the functionality of NPs has opened a new way to relevant therapeutic innovations. Future years, through the continuous efforts of several research groups, will certainly reveal more insights on this multifaceted cardiovascular hormonal system.

## References

[1] Levin E.R., Gardner D.G., Samson W.K. (1998). Natriuretic peptides. N. Engl. J. Med..

[2] Rubattu S., Sciarretta S., Valenti V., Stanzione R., Volpe M. (2008). Natriuretic peptides: An update on bioactivity, potential therapeutic use and implication in cardiovascular diseases. Am. J. Hypertens..

[3] Volpe M., Rubattu S., Burnett J. (2014). Natriuretic peptides in cardiovascular diseases: Current use and perspectives. Eur. Heart J..

[4] Das B.B., Solinger R. (2009). Role of natriuretic peptide family in cardiovascular medicine. Cardiovasc. Hematol. Agents Med. Chem..

[5] Volpe M., Tocci G., Battistoni A., Rubattu S. (2015). Angiotensin II receptor blocker nephrilysin inhibitor (ARNI): New avenues in cardiovascular therapy. High Blood Press. Cardiovasc. Prev..

[6] Kario K. (2018). The Sacubitril/Valsartan, a first-in-class, angiotensin receptor neprilysin inhibitor (ARNI): Potential uses in hypertension, heart failure, and beyond. Curr. Cardiol. Rep..

[7] Forte M., Madonna M., Schiavon S., Valenti V., Versaci F., Biondi Zoccai G., Frati G., Sciarretta S. (2019). Cardiovascular pleiotropic effects of atrial natriuretic peptide. Int. J. Mol. Sci..

[8] Pandey K.N. (2019). Genetic ablation and guanylyl cyclase/natriuretic peptide receptor A: Impact on the pathophysiology of cardiovascular dysfunction. Int. J. Mol. Sci..

[9] Rubattu S., Sciarretta S., Volpe M. (2014). Atrial natriuretic peptide gene variants and circulating levels: Implications in cardiovascular diseases. Clin. Sci. Lond.

[10] Lafontan M., Moro C., Berlan M., Crampes F., Sengenes C., Galitzky J. (2008). Control of lipolysis by natriuretic peptides and cyclic GMP. Trends Endocrinol. Metab..

[11] Bordicchia M., Liu D., Amri E.Z., Ailhaud G., Dessì-Fulgheri P., Zhang C., Takahashi N., Sarzani R., Collins S. (2012). Cardiac natriuretic peptides act via p38 MAPK to induce the brown fat thermogenic program in mouse and human adipocytes. J. Clin. Invest..

[12] Bordicchia M., Spannella F., Ferretti G., Bacchetti T., Vignini A., Di Pentima C., Mazzanti L., Sarzani R. (2019). PCSK9 is Expressed in Human Visceral Adipose Tissue and Regulated by Insulin and Cardiac Natriuretic Peptides. Int. J. Mol. Sci..

[13] Lagace T.A. (2014). PCSK9 and LDLR degradation: Regulatory mechanisms in circulation and in cells. Curr. Opin. Lipidol..

[14] Urban D., Pöss J., Böhm M., Laufs U. (2013). Targeting the proprotein convertase subtilisin/kexin type 9 for the treatment of dyslipidemia and atherosclerosis. J. Am. Coll. Cardiol..

[15] Sabatine M.S., Giugliano R.P., Keech A.C., Honarpour N., Wiviott S.D., Murphy S.A., Kuder J.F., Wang H., Liu T., Wasserman S.M. (2017). Evolocumab and Clinical Outcomes in Patients with Cardiovascular Disease. N. Engl. J. Med..

[16] Tomlinson B., Hu M., Zhang Y., Chan P., Liu Z.M. (2017). Alirocumab for the treatment of hypercholesterolemia. Expert Opin. Biol. Ther..

[17] Ibrahim N.E., McCarthy C.P., Shrestha S., Gaggin H.K., Mukai R., Szymonifka J., Apple F.S., Burnett J.C., Iyer S., Januzzi J.L. (2019). Effect of neprilysin inhibition on various natriuretic peptide assays. J. Am. Coll. Cardiol..

[18] Rubattu S., Forte M., Marchitti S., Volpe M. (2019). Molecular implications of natriuretic peptides in the protection from hypertension and target organ damage development. Int. J. Mol. Sci..

[19] Rubattu S., Sciarretta S., Marchitti S., Bianchi F., Forte M., Volpe M. (2018). The T2238C atrial natriuretic peptide molecular variant and the risk of cardiovascular diseases. Int. J. Mol. Sci..

[20] Cannone V., Scott C.G., Decker P.A., Larson N.B., Palmas W., Taylor K.D., Wang T.J., Gupta D.K., Bielinski S.J., Burnett J. (2017). A favorable cardiometabolic profile is associated with the G allele of the genetic variant rs5068 in African Americans: The Multi-Ethnic Study of Atherosclerosis (MESA). PLoS ONE.

[21] Rubattu S., Bigatti G., Evangelista A., Lanzani C., Stanzione R., Zagato L., Manunta P., Marchitti S., Venturelli V., Bianchi G. (2006). Association of atrial natriuretic and type-A natriuretic peptide receptor gene polymorphisms with left ventricular mass in human essential hypertension. J. Am. Coll. Cardiol..

[22] Maisel A.S., Krishnaswamy P., Nowak R.M., McCord J., Hollander J.E., Duc P., Omland T., Storrow A.B., Abraham W.T., Wu A.H. (2002). Breathing Not Properly Multinational Study Investigators. Rapid measurement of B-type natriuretic peptide in the emergency diagnosis of heart failure. N. Engl. J. Med..

[23] Mukoyama M., Nnakao K., Saito Y., Ogawa Y., Hosoda K., Suga S., Shirakami G., Jougasaki M., Imura H. (1990). Increased human brain natriuretic peptide in congestive heart failure. N. Engl. J. Med..

[24] Cui K., Huang W., Fan J., Lei H. (2018). Midregional pro-atrial natriuretic peptide is a superior biomarker to N-terminal pro-B-type natriuretic peptide in the diagnosis of heart failure patients with preserved ejection fraction. Med. Baltim..

[25] Maisel A.S., Duran J.M., Wettersten N. (2018). Natriuretic peptides in heart failure: Atrial and B-type natriuretic peptides. Heart Fail. Clin..

[26] Seronde M.F., Gayat E., Logeart D., Lassus J., Laribi S., Boukef R., Sibellas F., Launay J.M., Manivet P., Sadoune M. (2013). Comparison of the diagnostic and prognostic values of B-type and atrial-type natriuretic peptides in acute heart failure. Int. J. Cardiol..

[27] Lam C.S.P., Gamble G.D., Ling L.H., Sim D., Leong K.T.G., Yeo P.S.D., Ong H.Y., Jaufeerally F., Ng T.P., Cameron V.A. (2018). Mortality associated with heart failure with preserved vs reduced ejection fraction in a prospective multi-ethnic cohort study. Eur. Heart J..

[28] Volpe M., Rubattu S., Dorobantu M., Mancia G., Grassi G., Voicu V. (2019). Natriuretic peptides. Hypertension and Heart Failure.

[29] Tanase D.M., Rdu S., Al Shurbaji S., Baroi G.L., Costea C.L., Turliuc M.D., Ouatu A., Floria M. (2019). Natriuretic peptides in heart failure with preserved left ventricular ejection fraction: From molecular evidences to clinical implications. Int. J. Mol. Sci..

[30] Brunner-La Rocca H.P., Sanders-van Wijk S. (2019). Natriuretic peptides in chronic heart failure. Card. Fail. Rev..

[31] Solomon S.D., Rizkala A.R., Gong J., Wang W., Anand I.S., Ge J., Lam C.S.P., Maggioni A.P., Martinez F., Packer M. (2017). Angiotensin receptor neprilysin inhibition in heart failure with preserved ejection fraction: Rationale and design of the PARAGON-HF trail. JACC Heart Fail..

[32] Ellinor P.T., Low A.F., Patton K.K., Shea M.A., Macrae C.A. (2005). Discordant atrial natriuretic peptide and brain natriuretic peptide levels in lone atrial fibrillation. J. Am. Coll. Cardiol..

[33] Baba M., Yoshida K., Ieda M. (2019). Clinical application of natriuretic peptides in heart failure and atrial fibrillation. Int. J. Mol. Sci..

[34] Richards M., Di Somma S., Mueller C., Nowak R., Peacock W.F., Ponikowski P., Mockel M., Hogan C., Wu A.H., Clopton P. (2013). Atrial fibrillation impairs the diagnostic performance of cardiac natriuretic peptides in dyspneic patients: Results from the BACH Study (Biomarkers in Acute Heart Failure). JACC Heart Fail..

[35] Knudsen C.W., Omland T., Clopton P., Westheim A., Wu A.H., Duc P., McCord J., Nowak R.M., Hollander J.E., Storrow A.B. (2005). Impact of atrial fibrillation on the diagnostic performance of cardiac natriuretic peptide concentration in dyspneic patients: An analysis from the breathing not properly multinational study. J. Am. Coll. Cardiol..

[36] Beck-da-Silva L., de Bold A., Fraser M., Williams K., Haddad H. (2004). Brain natriuretic peptide predicts successful cardioversion in patients with atrial fibrillation and maintenance of sinus rhythm. Can. J. Cardiol..

[37] Deng H., Shantsila A., Guo P., Zhan X., Fang X., Liao H., Liu Y., Wei W., Fu L., Wu S. (2018). Multiple biomarkers and arrhythmia outcome following catheter ablation of atrial fibrillation: The Guangzhou Atrial Fibrillation Project. J. Arrhytm..

[38] Okamoto R., Ali Y., Hashizume R., Suzuki N., Ito M. (2019). BNP as a major player in the heart-kidney connection. Int. J. Mol. Sci..

[39] Dos Reis D., Fraticelli L., Bassand A., Manzo-Silberman S., Peschanski N., Charpentier S., Elbaz M., Savary D., Bonnefoy-Cudraz E., Laribi S. (2019). Impact of renal dysfunction on the management and outcome of acute heart failure: Results from the French prospective, multicenter, DeFSSICA survey. BMJ Open.

[40] McKie P.M., Schirger J.A., Benike S.L., Hastad L.K., Slusser J.P., Hodge D.O., Redfield M.M., Burnett J.C., Chen H.H. (2016). Chronic subcutaneous brain natriuretic peptide therapy in asymptomatic systolic heart failure. Eur. J. Heart Fail..

[41] Cao Z., Jia Y., Zhu B. (2019). BNP and NT-proBNP as Diagnostic Biomarkers for Cardiac Dysfunction in Both Clinical and Forensic Medicine. Int. J. Mol. Sci..

[42] Horio T., Tokudome T., Maki T., Yoshihara F., Suga S., Nishikimi T., Kojima M., Kawano Y., Kangawa K. (2003). Gene expression, secretion, and autocrine action of C-type natriuretic peptide in cultured adult rat cardiac fibroblasts. Endocrinology.

[43] Moyes A., Hobbs A. (2019). C-Type Natriuretic Peptide: A Multifaceted Paracrine Regulator in the Heart and Vasculature. Int. J. Mol. Sci..

[44] Nakao K., Kuwahara K., Nishikimi T., Nakagawa Y., Kinoshita H., Minami T., Kuwabara Y., Yamada C., Yamada Y., Tokudome T. (2017). Endothelium-Derived C-Type Natriuretic Peptide Contributes to Blood Pressure Regulation by Maintaining Endothelial Integrity. Hypertension.

[45] Del Ry S., Passino C., Maltinti M., Emdin M., Giannessi D. (2005). C-type natriuretic peptide plasma levels increase in patients with chronic heart failure as a function of clinical severity. Eur. J. Heart Fail..

[46] Egom E.E.A., Feridooni T., Pharithi R.B., Khan B., Shiwani H.A., Maher V., El Hiani Y., Rose R.A., Pasumarthi K.B.S., Ribama H.A. (2017). New insights and new hope for pulmonary arterial hypertension. Int. J. Physiol. Pathophysiol. Pharmacol..

[47] Egom E.E.A. (2019). Pulmonary arterial hypertension due to NPR-C mutation: A novel paradigm for normal and pathologic remodeling?. Int. J. Mol. Sci..

[48] Li Y., Hashim S., Anand-Srivastava M.B. (2006). Intracellular peptides of natriuretic peptide receptor-C inhibit vascular hypertrophy via Gqa/MAP kinase signaling pathways. Cardiovasc. Res..

[49] Cannone V., Cabassi A., Volpi R., Burnett J.C. (2019). Atrial natriuretic peptide: A molecular target of novel therapeutic approaches to cardio-metabolic disease. Int. J. Mol. Sci..

[50] Volpe M., Rubattu S., Battistoni A. (2019). ARNi: A novel approach to counteract cardiovascular diseases. Int. J. Mol. Sci..

